# Supplementary material for: Genetic and environmental determinants of multicellular-like phenotypes in fission yeast
Source: Genetics. 2026 Mar 6;233(1):iyag064. doi: 10.1093/genetics/iyag064 (PMC13147542; doi:10.1093/genetics/iyag064)
Supplement: iyag064_Supplementary_Data [file iyag064_supplementary_data.zip › Supplemental_Material_GENETICS-2026-309023.pdf]

Supplemental Figure 1

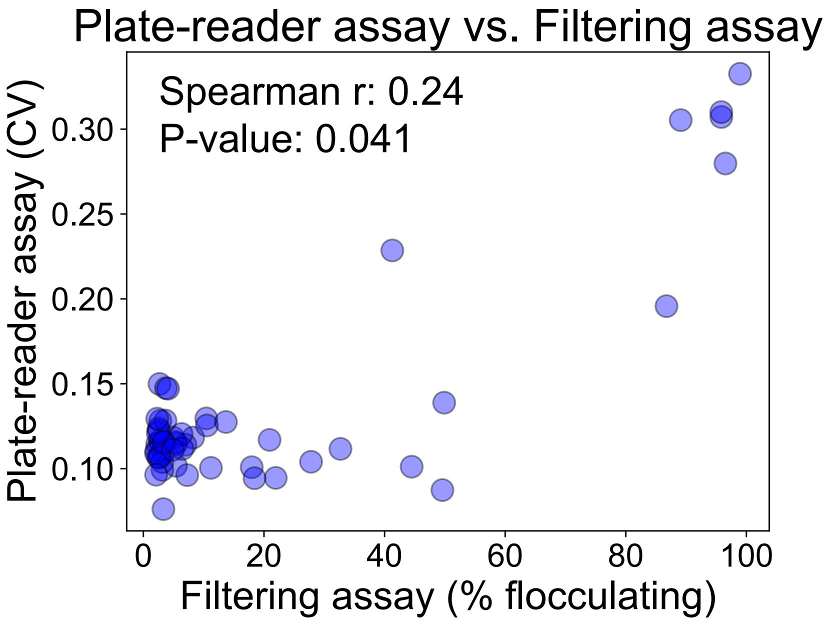

Supplemental Figure 2

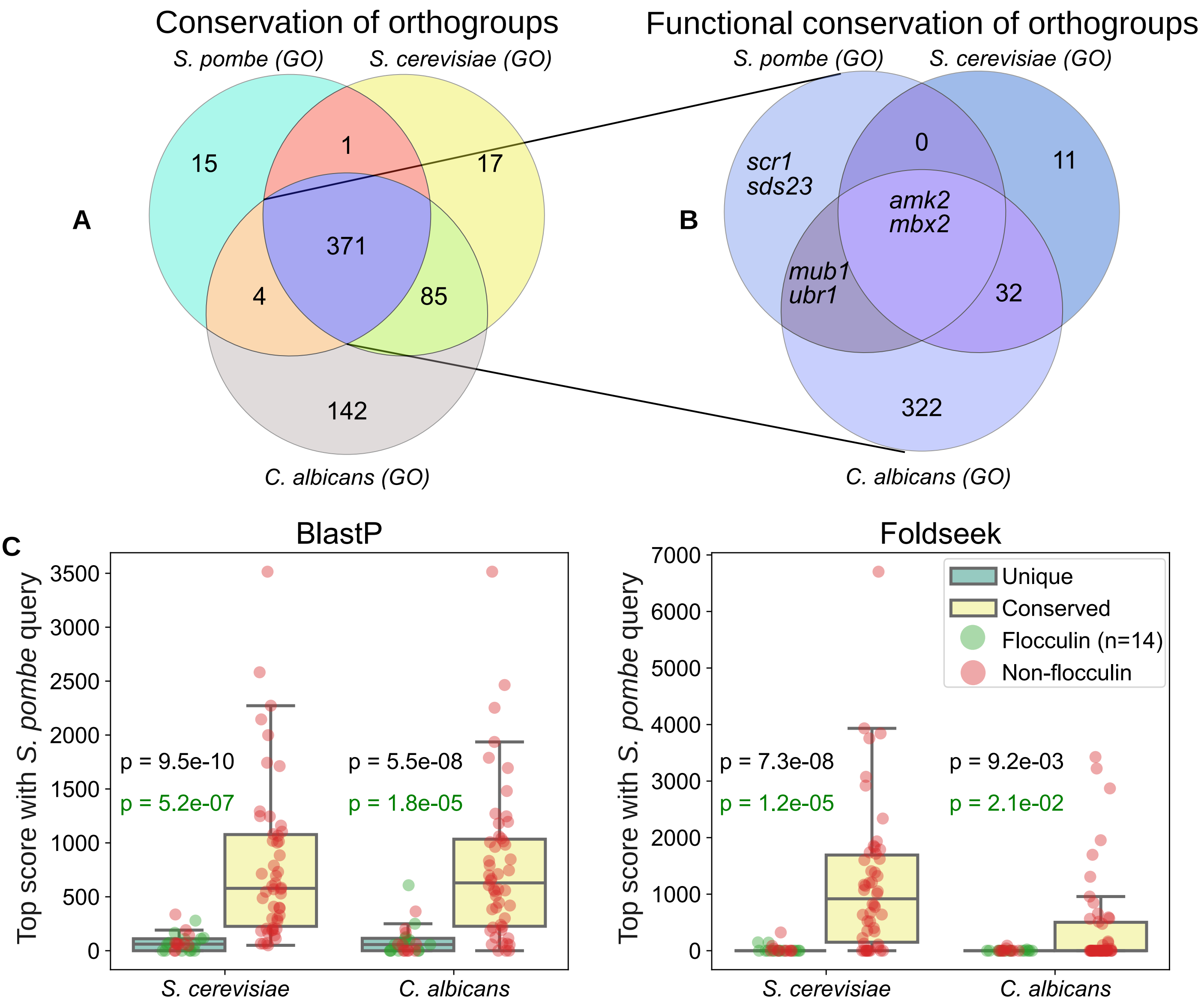

Supplemental Figure 3

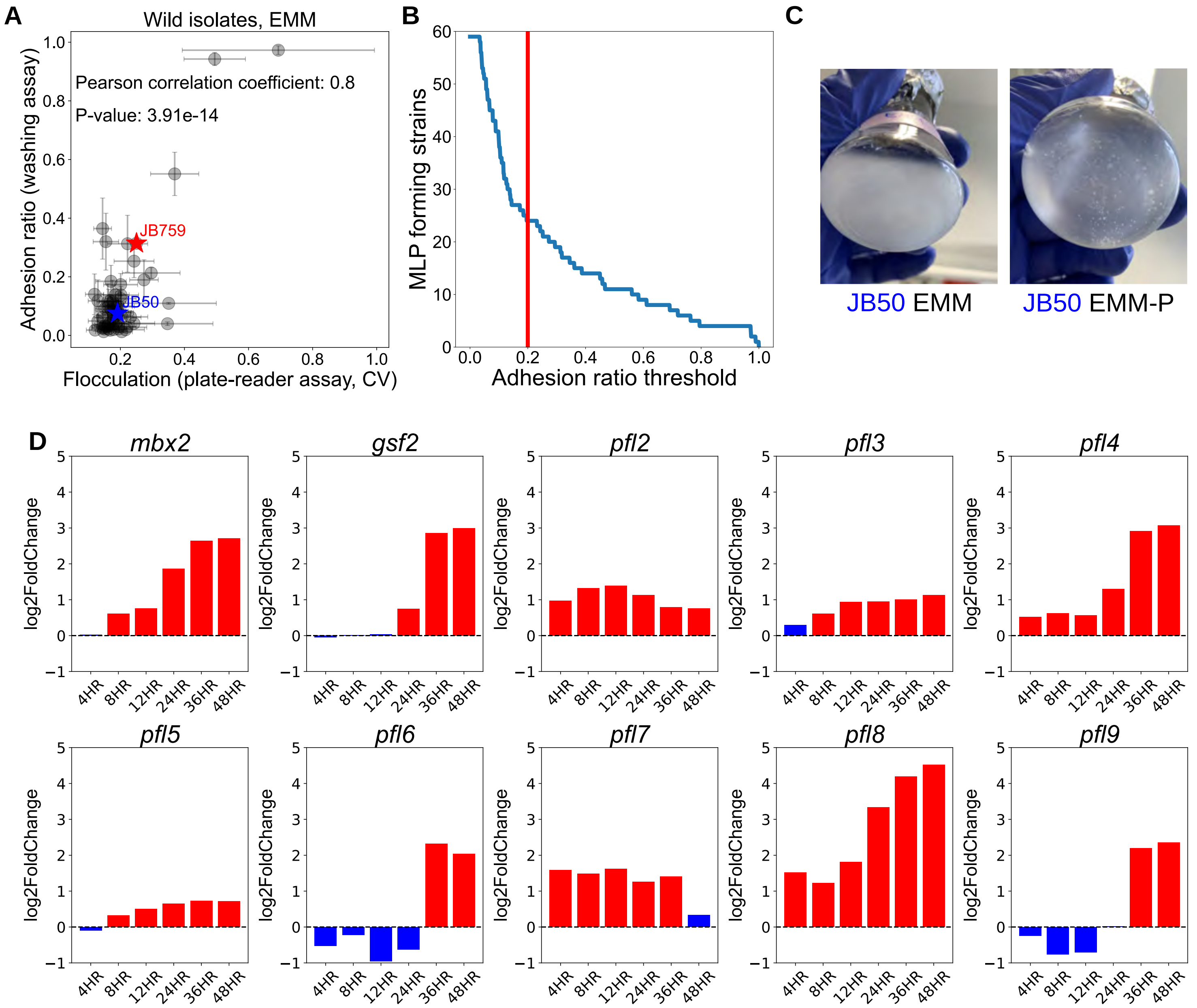

Supplemental Figure 4

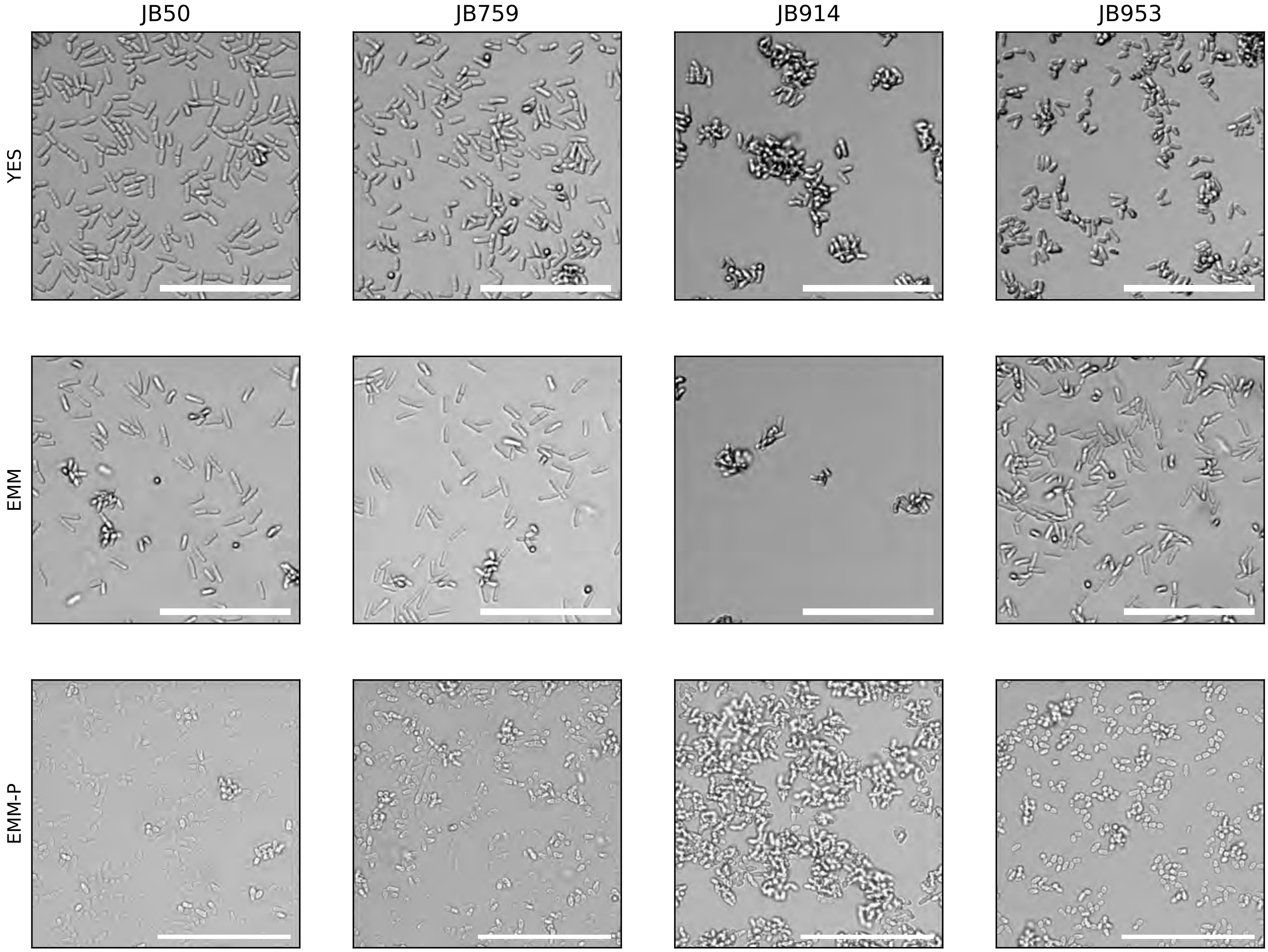

Supplemental Figure 5

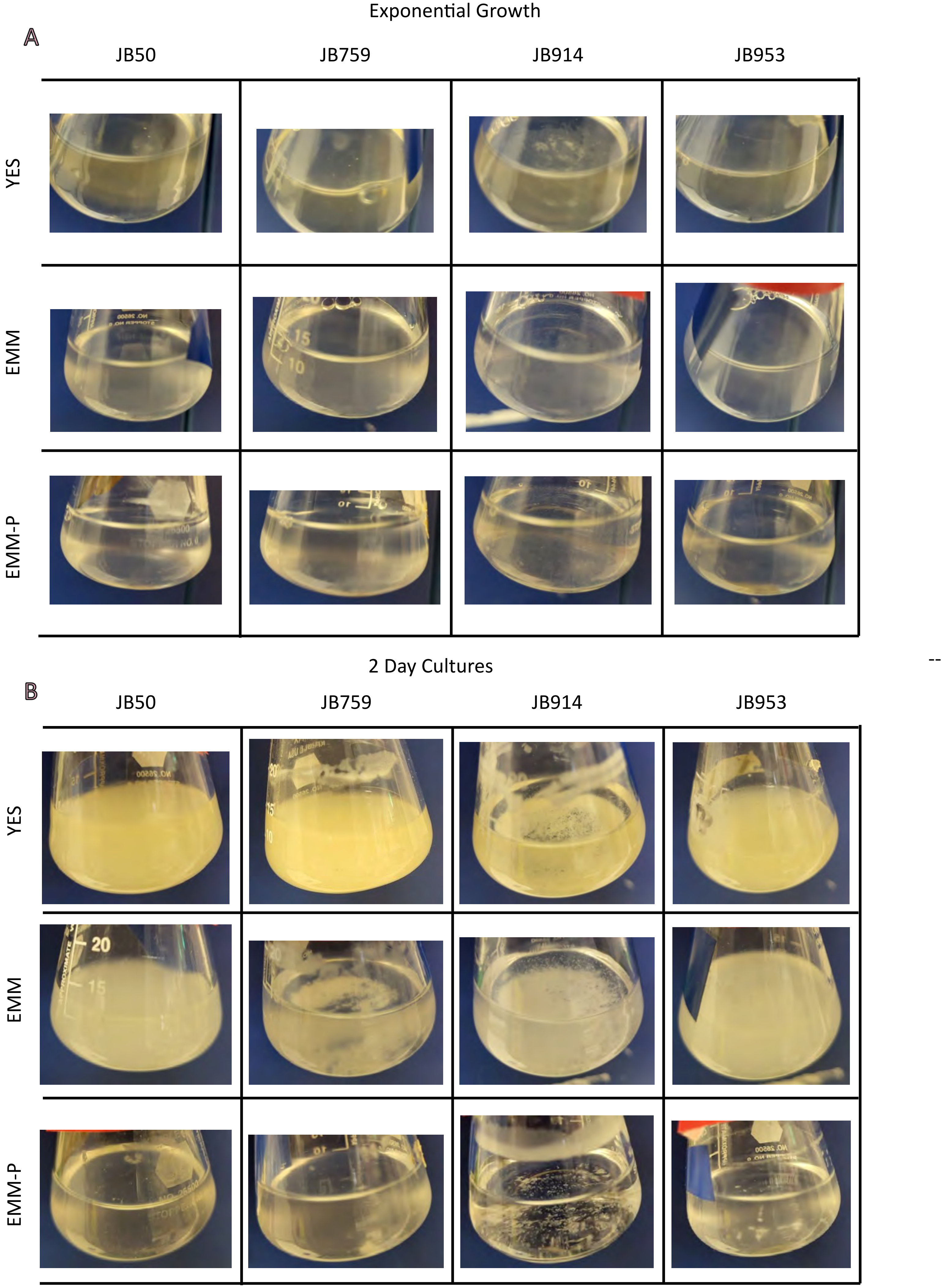

Supplemental Figure 6

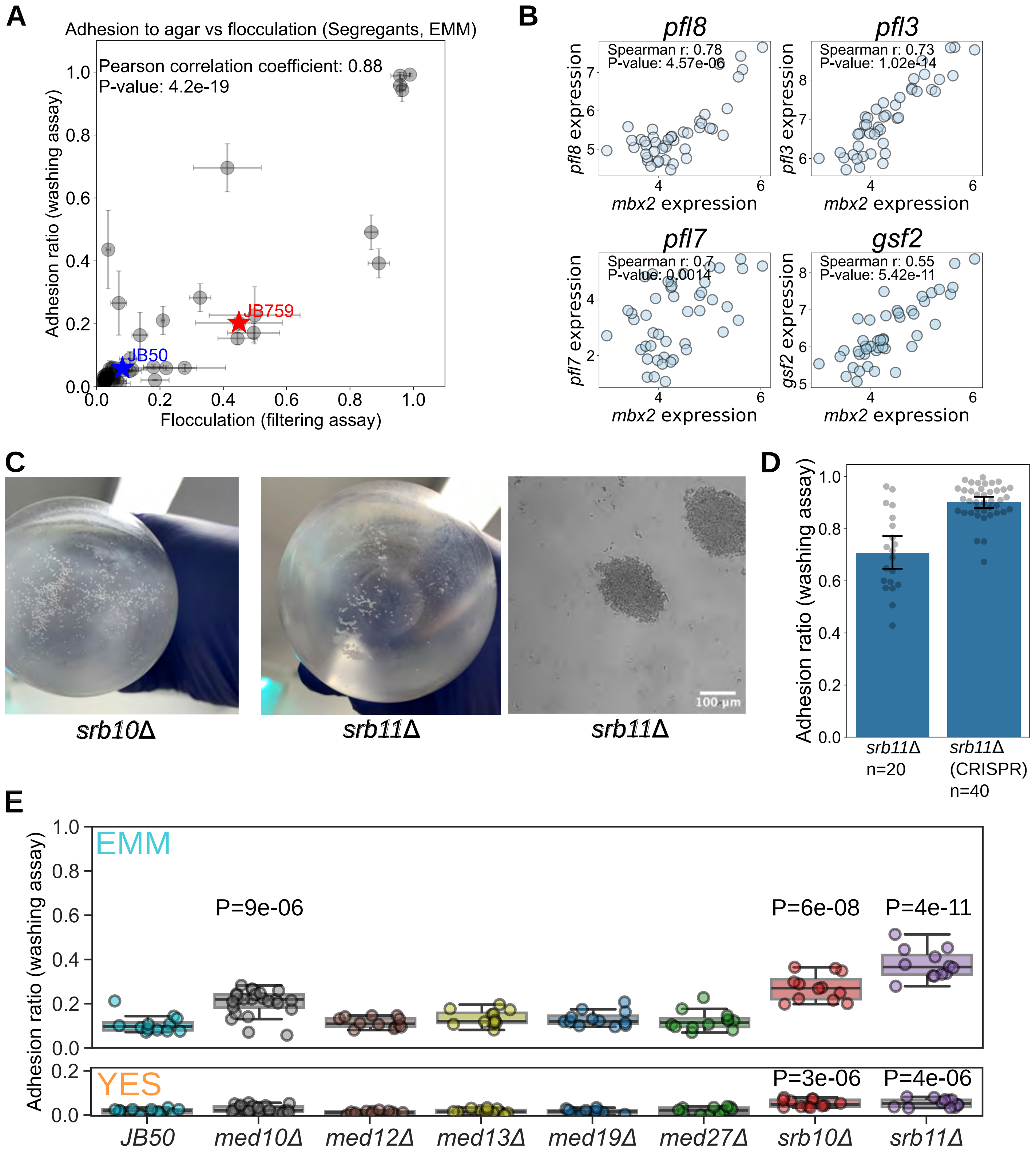

Supplemental Figure 7

A

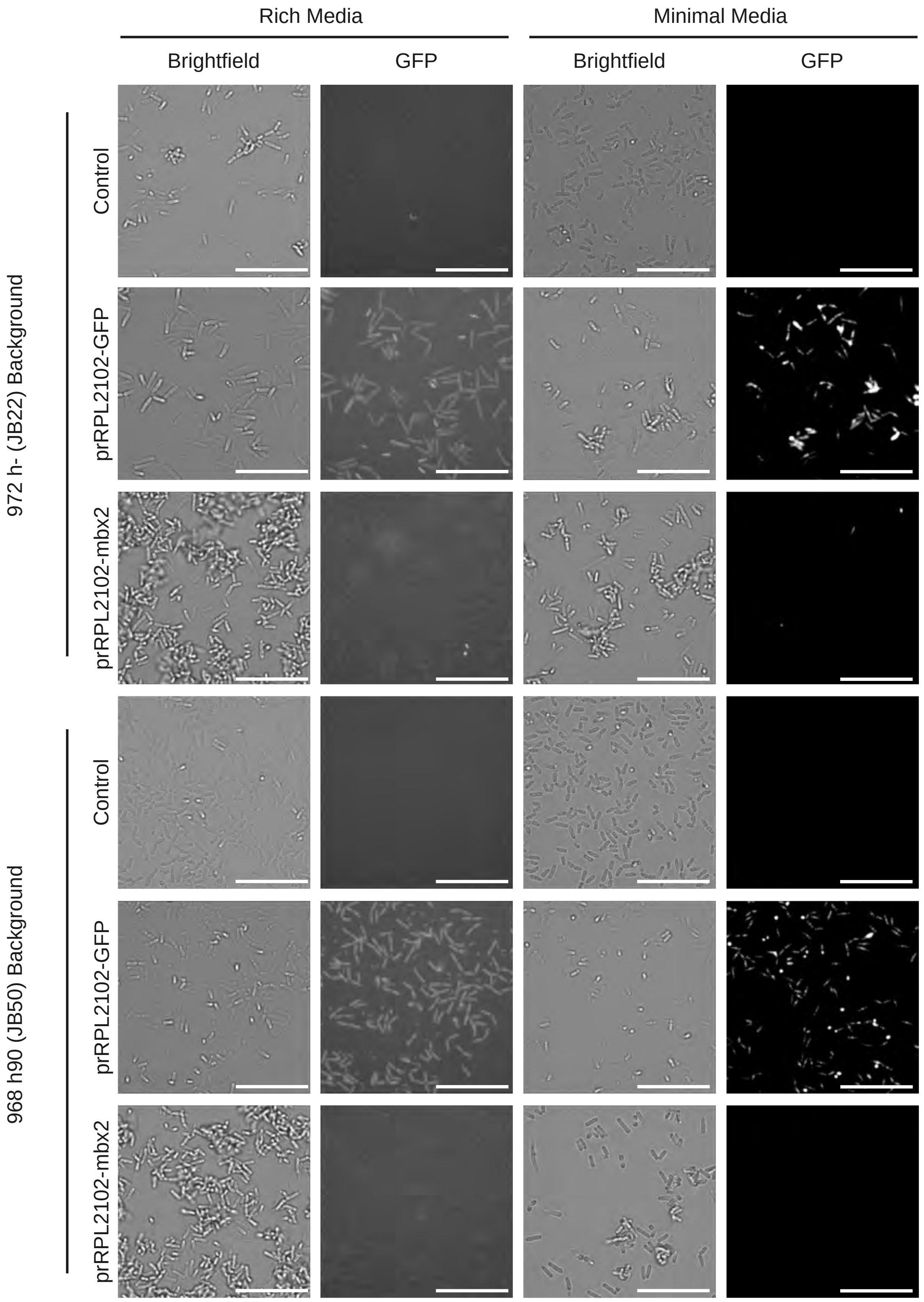

B

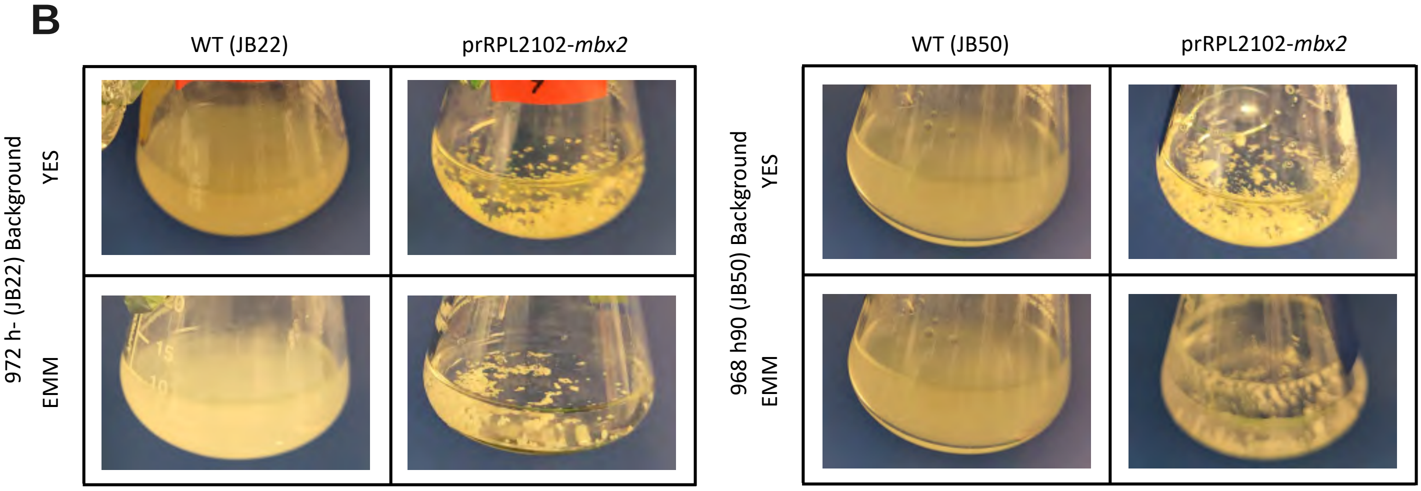

Supplemental Figure 8

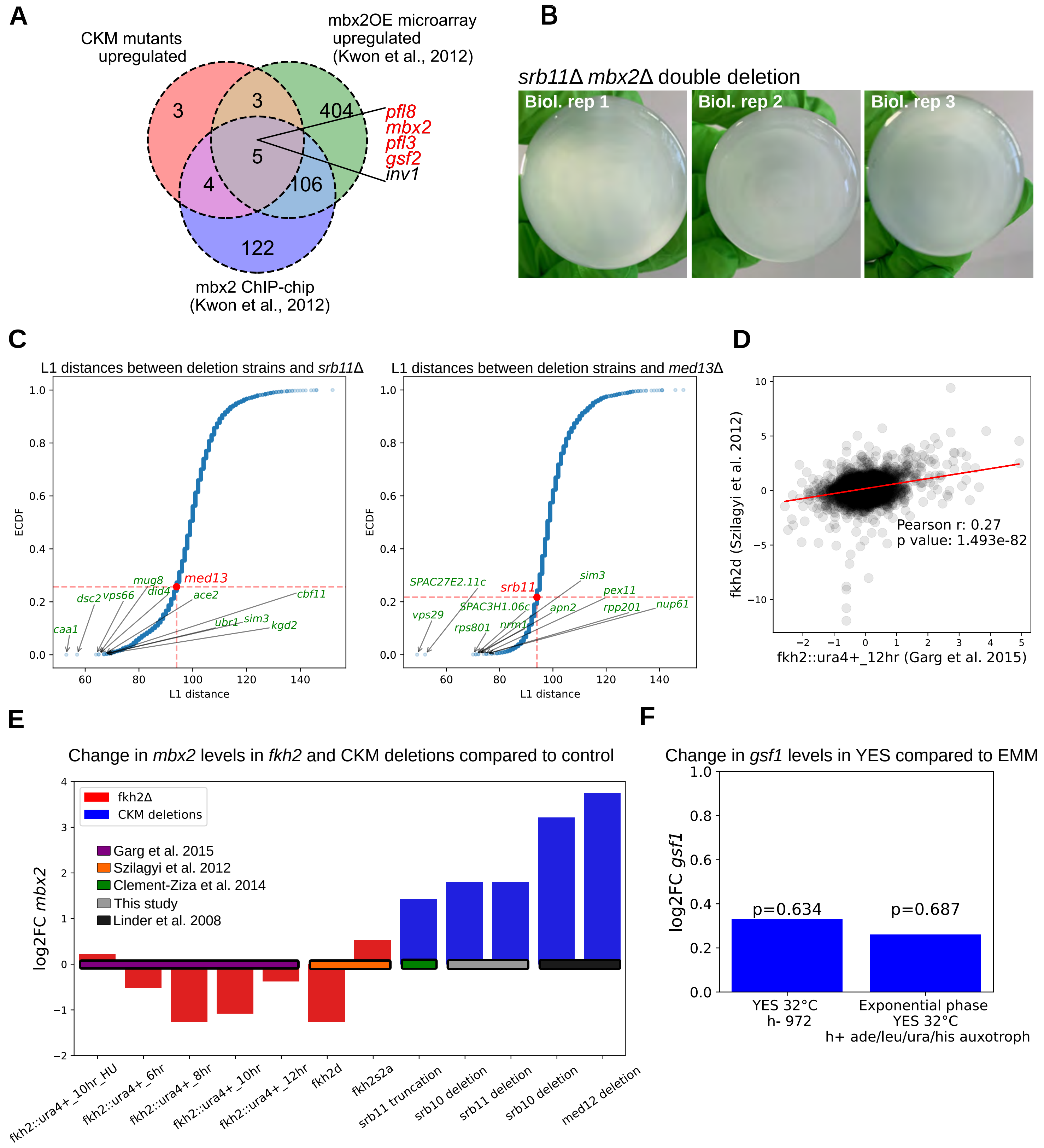

Supplemental Figure 9

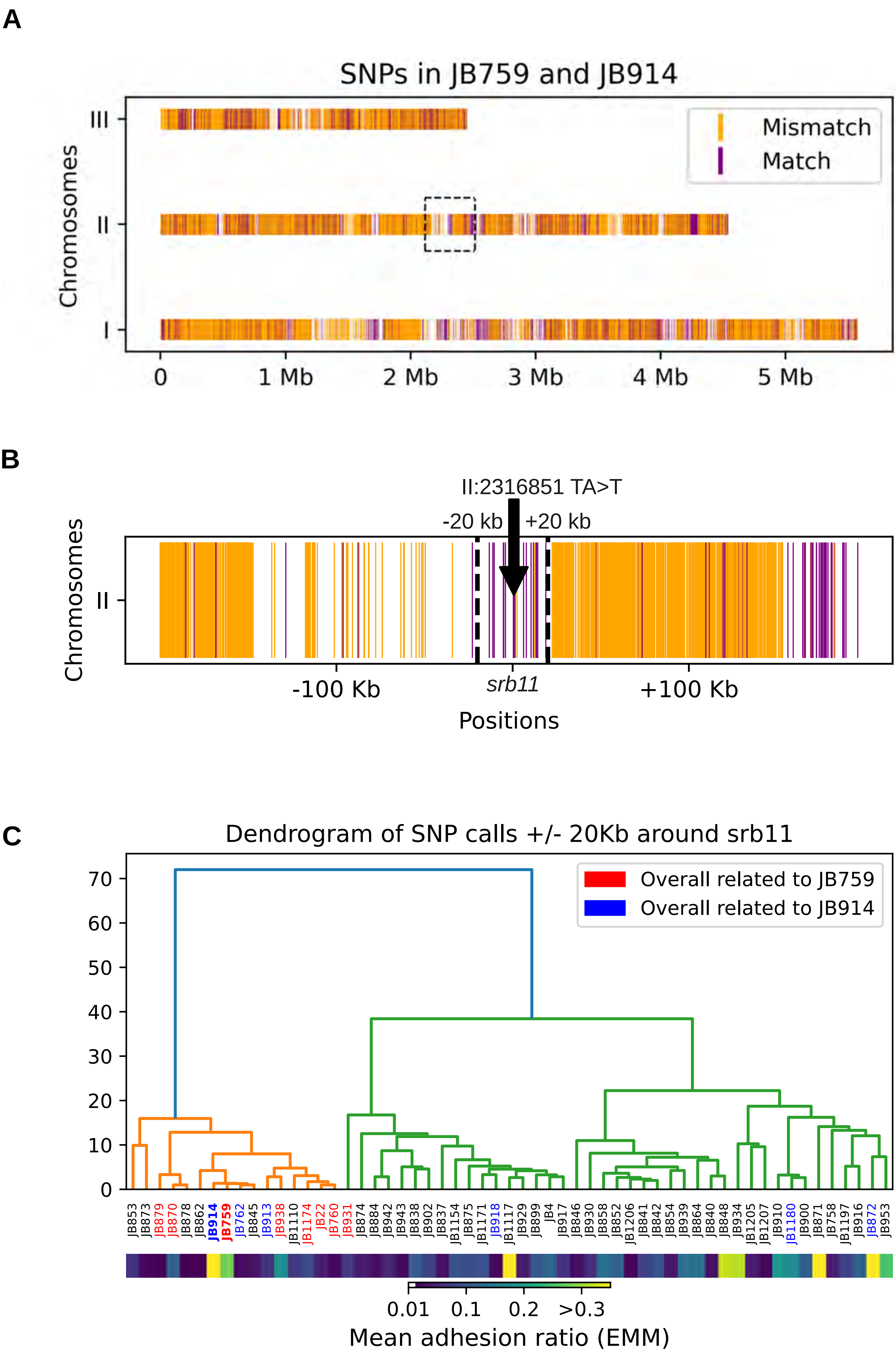

Supplemental Figure 10

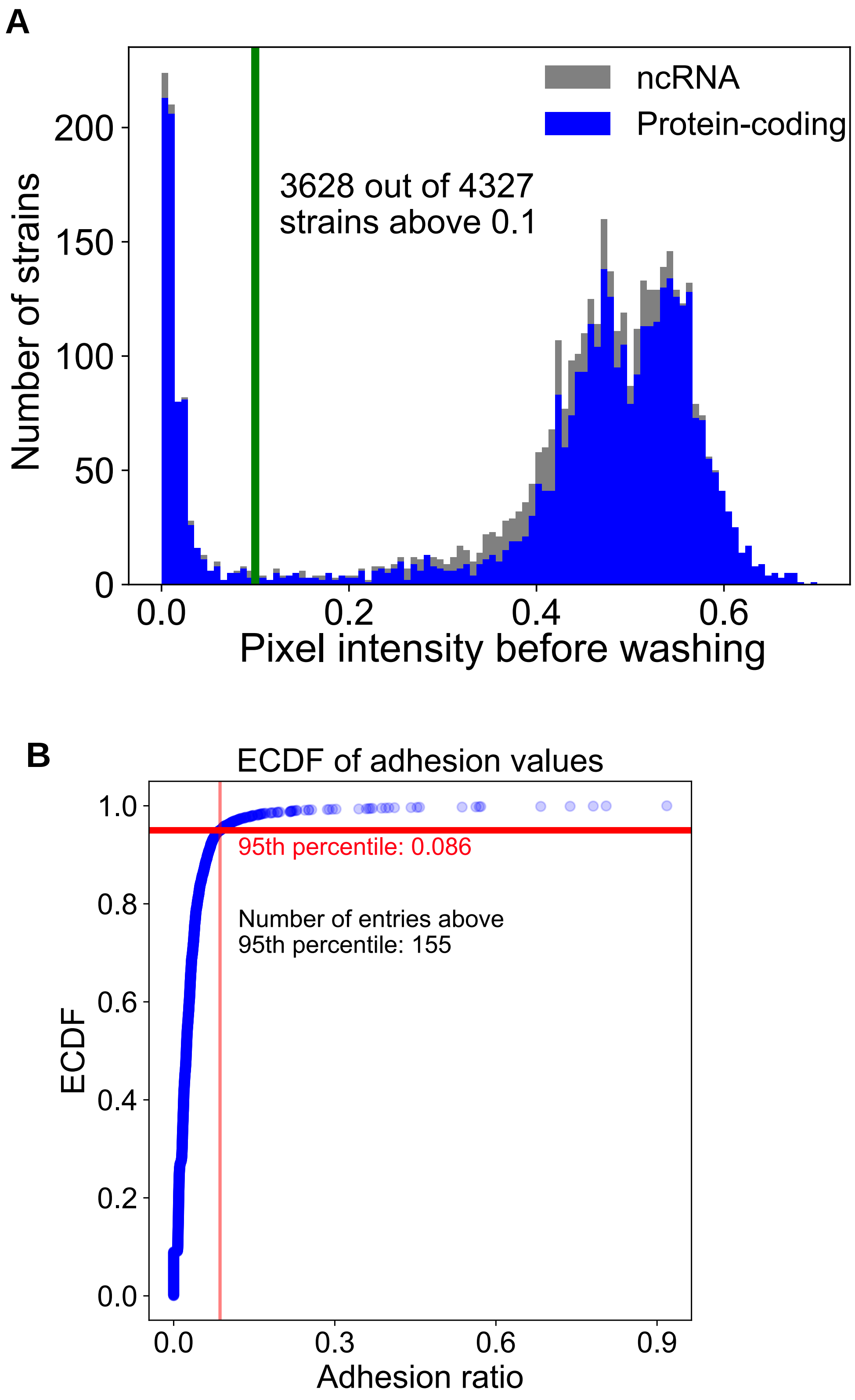

Supplemental Figure 11

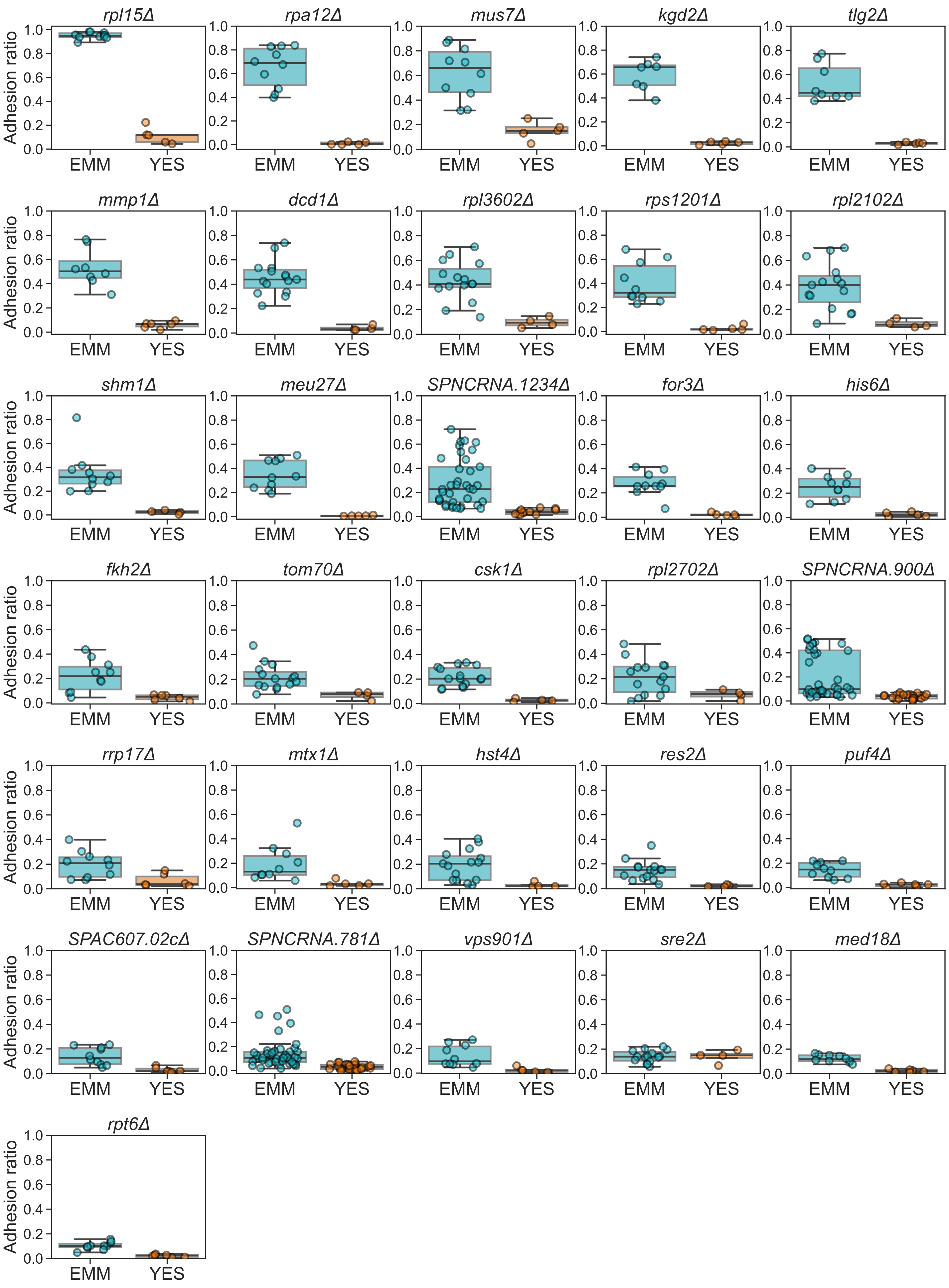

Supplemental Figure 12

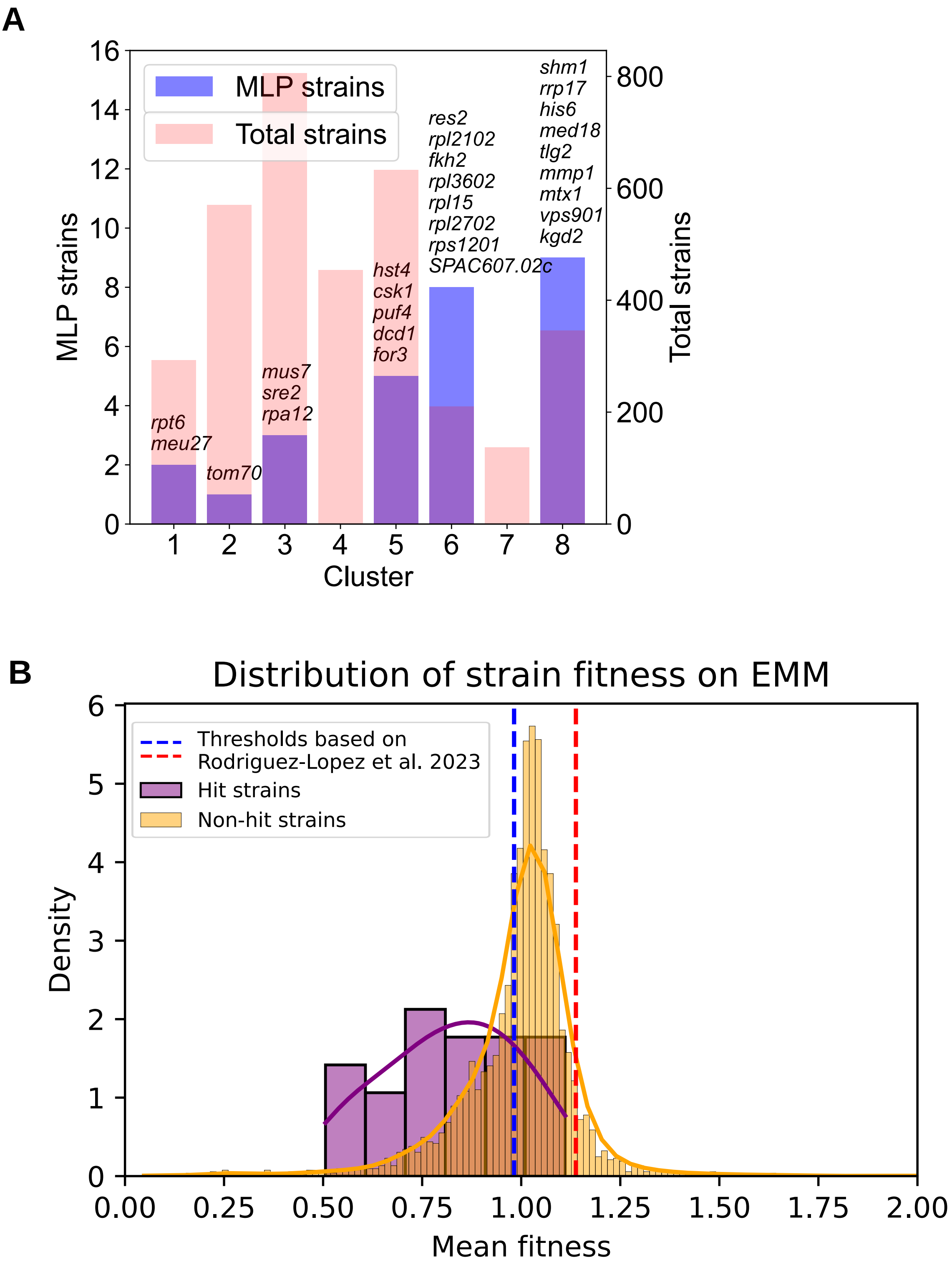

Supplemental Figure Legends

Supplemental Figure 1: Plate-reader assay and filtering assay for flocculation are correlated.

Scatter plot comparison of flocculation for the JB50-JB759 segregant library by measuring each strain with a conventional filtering assay (x-axis, n=3) and our high-throughput plate-reader assay which measures the CV of OD600 within each well (y-axis, n=5) (Methods). Each point represents the mean flocculation values for a strain. P-value was obtained with a one-tailed test on whether the Spearman correlation coefficient is larger than 0.

Supplemental Figure 2: Conservation of MLP-related orthogroups between *S. pombe*, *S. cerevisiae* and *C. albicans*.

(A) Venn diagram representing genetic and (B) functional conservation of orthogroups between the three species using data only from GO-terms. (C) Highest alignment scores for either 25 protein sequences annotated as unique to *S pombe* or 50 random conserved sequences using either BLAST-P or Foldseek to identify similar proteins in the *S. cerevisiae* or *C. albicans* proteome (Methods). Each dot represents the highest score using the indicated search algorithm for a given protein. P-values were derived from Mann-Whitney U tests comparing top scores for conserved proteins with either all unique proteins (black) or just unique *S. pombe* flocculins (green).

Supplemental Figure 3: Further analysis on adhesion phenotypes of the natural isolates across different conditions.

(A) Scatter plot comparison of flocculation and adhesion for the natural isolate library by measuring each strain with a high-throughput plate-reader assay (x-axis, n=11) and our high-throughput adhesion assay (y-axis, n=10). Each point represents the mean values for a strain with the standard error of the mean as error bars. Stars mark JB759 (red) and the lab strain JB50 (blue). (B) Elbow plot of the number of strains designated as "MLP-forming" in at least one condition (y-axis) given different cut-off thresholds (x-axis). Empirically we found that 0.2 sits in the elbow of the plot, suggesting that it is a reasonable cut-off. (C) Phosphate starvation induces MLP formation in the lab strain JB50. Images of the JB50 strain growing in 25ml flasks with 10ml media of either EMM (left) or EMM-P (right). Strains were first grown up on YES plates and then transferred to liquid cultures. There are clearly visible flocs forming in EMM-P. (D) Bar plot of log2FC in gene expression during 2 days of phosphate starvation for selected genes, including the transcription factor *mbx2* and various flocculins. Data from (Garg *et al.*, 2023). Red bars represent significant log2FC values.

Supplemental Figure 4: Microscope images of wild isolates exhibiting MLPs.

Images of during exponential growth of JB50 (lab strain) and three wild isolates (JB759, JB914, and JB953) in media conditions under which they exhibit MLPs. Note: EMM-P images were taken with a 20x objective with 1x zoom, while EMM and YES images were taken with a 5x objective and 2x optical zoom. Images were scaled to be comparable. The images of JB914 and JB953 in EMM and YES are duplicated from this panel and also included in Figure 2. Scale bars are 100 µm.

Supplemental Figure 5: Culture flask images of wild isolates exhibiting MLPs.
Images of during (A) exponential growth and (B) in saturated cultures after two days of JB50 (lab strain) and three wild isolates (JB759, JB914, and JB953) in media conditions under which they exhibit MLPs.

Supplemental Figure 6: MLP formation in *S. pombe* involves *mbx2* and CKM components.
(A) Scatter plot comparison of flocculation and adhesion for the JB50-JB759 library by measuring each strain with the filtering assay (x-axis, n=3) and the high-throughput adhesion assay (y-axis, n=10). Each point represents the mean values for a strain with the standard error of the mean as error bars. Stars mark the two parental strains JB759 (red) and JB50 (blue). There is a strong correlation between the two MLPs. (B) Scatter plot of expression levels of four flocculin genes against *mbx2* expression in the JB50-JB759 segregant library (data from (Clément-Ziza *et al.*, 2014)), demonstrating a strong association. (C) (Left) Images showing flocculating cultures of *srb10* and *srb11* in 250ml flasks grown in EMM. (Right) Representative microscopy image of *srb11*Δ::Kan (D) Barplot of adhesion values showing that the Kan deletion of *srb11* found in the deletion library phenocopies the CRISPR deletion of *srb11*. Each dot is an independent measurement. Error bars represent the 95% confidence interval for the mean. (E) Box plot showing adhesion values from validation cohort of fresh Mediator gene deletion strains on EMM and YES as indicated. Each dot represents a replicate.

Supplemental Figure 7: Overexpression of *mbx2* drives MLP formation.
(A) Microscopy and (B) flask images illustrating the effects of *mbx2* overexpression on cells grown in EMM or YES for both the h- (JB22) and h90 (JB50) backgrounds and the h90 JB50 background. Flask images were from overnight cultures, and microscope images were from overnight cultures after dilution into fresh media for two hours. The flask images of prRPL2102-*mbx2* (JB22) are duplicated and also shown in Figure 3. Scale bars are 75 µm.

Supplemental Figure 8: Mbx2 drives MLP formation in the *srb11Δ* strain.
(A) Venn diagram showing the intersection of three gene sets: (i) Genes upregulated in CKM mutants, meaning upregulated in the *med12Δ* and *srb10Δ* microarray data from (Linder *et al.*, 2008) and in the *srb11* truncated segregant RNA-seq data from (Clément-Ziza *et al.*, 2014) (Fig 4E), (ii) Genes

upregulated after *mbx2* overexpression obtained from (Kwon *et al.*, 2012) and (iii) Genes bound by Mbx2 obtained from (Kwon *et al.*, 2012). (B) Images showing non-flocculating cultures of three biological replicates of *srb11/mbx2* double deletion strains. (C) Empirical cumulative distribution function of L1 distances between phenotype vectors for the *srb11Δ* strain and all other gene deletion strains for growth phenotypes across 131 different conditions. For each condition in each strain, a number of -1, 0, or 1 was assigned to denote sensitivity, neutrality and resistance, and the L1 distance is the sum of the absolute value of the differences across all phenotypes as measured in (Rodríguez-López *et al.*, 2023). The 10 deletion strains closest in their phenotypes to *srb11Δ* and *med13Δ* are highlighted on the plot. The similarity score of *med13Δ* is in the 21st percentile. (D) Scatter plot of fold changes comparing two different data sources for transcriptomic changes upon *fkh2* deletion. Dots mark individual genes in the datasets, while the line of best fit is shown in red. This plot concludes that it is fair to use the two data sources to draw joint conclusions from them. (E) Bar plot showing log2 fold-changes in *mbx2* expression levels from experiments in which *fkh2* was deleted (red) and in which CKM subunits are deleted (blue). Selected *fkh2* deletion experiments included microarray data from (Szilagyi *et al.*, 2012) and (Garg, Futcher and Leatherwood, 2015), while CKM subunit deletion experiments included *med12Δ* and *srb10Δ* microarray data from (Linder *et al.*, 2008), fold change calculated by grouping RNA-seq data from (48) for segregants with the *srb11* truncation vs those without the mutation (Fig 3D), and the RT-qPCR data collected in this work. (F) Bar plot showing log2 fold-changes in *gsf1* expression in YES compared to EMM. There is no significant upregulation in either of the two replicates. Data and P-values were taken from (Atkinson *et al.*, 2018).

Supplemental figure 9: The *srb11* variant is present in the JB759 and JB914 natural isolates, and segregates with MLP-formation across natural isolates closely related to those strains.

(A) *S. pombe* reference genome visualized for each of the three chromosomes, with vertical lines marking variant loci (called in (Jeffares *et al.*, 2015)) compared to the reference genome in at least one of the two strains. Variant loci with the same alternative allele for JB759 and JB914 are marked with purple, while variant loci where alleles do not match are marked with orange. (B) Inset showing the region surrounding the causal *srb11* variant. The +/- 20Kb window is highlighted. Vertical lines indicate variant alleles as in A. (C) Dendrogram illustrating genomic similarity of natural isolates based on the +/- 20Kb window around the *srb11* frameshift mutation. Red text indicates strains closely related to JB759 considering the whole genome (based on (Jeffares *et al.*, 2015)), while blue text indicates strains related to JB914. Heatmap below indicates adhesion ratio in EMM.

Supplemental Figure 10: Deletion library screen for MLP formation on EMM.

(A) Histogram showing cell densities, measured by inverse pixel intensity after 4 days of growth, for strains in our deletion library screen before washing. (B) Empirical cumulative distribution function (ECDF) of adhesion ratios from the deletion library screen, with red lines showing the cut-off for the 95th percentile. Strains above the cut-off were used for enrichment analysis.

Supplemental Figure 11: Verified hits from the deletion library screen for MLP formation, assayed both on EMM and YES.
Box plots of adhesion ratios obtained with the washing assay for the 31 high-confidence hits on EMM (light blue) vs YES (orange). Each dot is an independent observation. Adhesion was generally specific to EMM, except in the case of *sre2Δ.* The images of *rpl15Δ*, *rpa12Δ*, *fkh2Δ*, *SPNCRNA.1234Δ* are duplicated from this panel and also included in Figure 5.

Supplemental Figure 12: MLP-forming hits belong in various phenotypic clusters, but generally exhibit slow growth.
(A) Bar plot showing the number of MLP-forming (blue, left axis scale) and total (blue, right axis scale) strains in each broad phenotypic cluster of deletion strains identified in (Rodríguez-López *et al.*, 2023). (B) Histogram of mean fitness values, based on growth in solid EMM media, for each strain as defined in (Rodríguez-López *et al.*, 2023). MLP-forming hit strains are shown in purple, and all other strains are in yellow. Blue and red dotted lines show the thresholds beyond which strains were defined as slow- or fast-growing, respectively.

List of Supplemental Tables

Supplemental Table 1: Summary of the GO terms, FYPO terms, and phenotype keywords used for the orthology analysis.

Supplemental Table 2: Summary of results from the structure and sequence homology analysis in a tidy format, ready for plotting.

Supplemental Table 3: Summary of omics data sources used in the study.

Supplemental Table 4: Table of segregant genotypes used in the QTL analysis. Y0036 is alternative name for JB759, while 968 is for JB50

Supplemental Table 5: Table of orthology relatonships for MLP-related genes. Each combination of orthology relationships is summarized by one of the 7 "orthopatterns", representative of the 7 sets in the plotted Venn diagrams.

Supplemental Table 6: Measurements of adhesion phenotypes in the natural isolates in a tidy format, assembled from the output of yeastmlp, ready for plotting.

Supplemental Table 7: Correlation analysis between flocculation (filtering assay) and gene expression (Clement-Ziza RNA-seq) data.

Supplemental Table 8: Adhesion ratios of mediator deletion strains, assembled from the output of yeastmlp.

Supplemental Table 9: Differentially expressed genes in the srb11 deletion strain from the output of DESeq2.

Supplemental Table 10: Raw results from the deletion screen as measured by yeastmlp.

Supplemental Table 11: Enrichment analysis based on the top 5 percent of adhesive strains identified in the deletion screen.

Supplemental Table 12: Final results from the 31 high-confidence strains, as measured by yeastmlp.

Supplemental Table 13: Variants identified in candidate genes (from screen and Mediator genes) by short variant analysis in MLP-forming wild isolates.

Supplemental Table 14: List of plasmids used in this study.

Supplemental Literature Cited

Atkinson, S.R. et al. (2018) “Long noncoding RNA repertoire and targeting by nuclear exosome, cytoplasmic exonuclease, and RNAi in fission yeast,” RNA, 24(9), pp. 1195–1213. Available at: https://doi.org/10.1261/rna.065524.118.

Clément-Ziza, M. et al. (2014) “Natural genetic variation impacts expression levels of coding, non-coding, and antisense transcripts in fission yeast,” Molecular Systems Biology, 10(11), p. 764. Available at: https://doi.org/10.15252/msb.20145123.

Garg, A. et al. (2023) “Cellular responses to long-term phosphate starvation of fission yeast: Maf1 determines fate choice between quiescence and death associated with aberrant tRNA biogenesis,” Nucleic Acids Research, 51(7), pp. 3094–3115. Available at: https://doi.org/10.1093/nar/gkad063.

Garg, A., Futcher, B. and Leatherwood, J. (2015) “A new transcription factor for mitosis: in Schizosaccharomyces pombe, the RFX transcription factor Sak1 works with forkhead factors to regulate mitotic expression,” Nucleic Acids Research, 43(14), pp. 6874–6888. Available at: https://doi.org/10.1093/nar/gkv274.

Jeffares, D.C. et al. (2015) “The genomic and phenotypic diversity of Schizosaccharomyces pombe,” Nature Genetics, 47(3), pp. 235–241. Available at: https://doi.org/10.1038/ng.3215.

Kwon, E.-J.G. et al. (2012) “Deciphering the Transcriptional-Regulatory Network of Flocculation in Schizosaccharomyces pombe,” PLoS Genetics, 8(12), p. e1003104. Available at: https://doi.org/10.1371/journal.pgen.1003104.

Linder, T. et al. (2008) “Two conserved modules of Schizosaccharomyces pombe Mediator regulate distinct cellular pathways,” Nucleic Acids Research, 36(8), pp. 2489–2504. Available at: https://doi.org/10.1093/nar/gkn070.

Rodríguez-López, M. et al. (2016) “A CRISPR/Cas9-based method and primer design tool for seamless genome editing in fission yeast,” Wellcome Open Research, 1, p. 19. Available at: https://doi.org/10.12688/wellcomeopenres.10038.3.

Szilagyi, Z. et al. (2012) “Cyclin-Dependent Kinase 8 Regulates Mitotic Commitment in Fission Yeast,” Molecular and Cellular Biology, 32(11), pp. 2099–2109. Available at: https://doi.org/10.1128/MCB.06316-11.
